# Feature Paper Special Issue for Editorial Board Members (EBMs) of *Diseases*

**DOI:** 10.3390/diseases10020018

**Published:** 2022-03-22

**Authors:** Maurizio Battino

**Affiliations:** 1 Department of Clinical Sciences, Polytechnic University of Marche, 60131 Ancona, Italy; m.a.battino@unipm.it; Tel.: +39-071-220-4146; 2 International Joint Research Laboratory of Intelligent Agriculture and Agri-Product Processing, Jiangsu University, Zhenjiang 212013, China; 3 Research Group on Foods, Nutritional Biochemistry and Health, Universidad Europea del Atlántico, 39011 Santander, Spain

When you are part of a community, especially a scientific one, you are required to contribute significantly to its welfare, because the community as a whole represents each individual within it and, in turn, determines the wellbeing of the participants themselves.

This is also the case of the Editorial Board of *Diseases*.

I had the pleasure, honor and privilege of being named Editor-in-Chief of this journal several years ago when it was in its infancy; it had few yearly submissions, a limited editorial board, and it was not considered to be consistent enough to be indexed in PubMed or the Web of Science database.

The hard work of the Editorial Office team and all Editorial Board colleagues, as well as the authors and reviewers who have contributed to the journal, has led *Diseases* to be considered a promising ESCI (Emerging Source Citation Index) journal by Clarivate/Web of Science and to be indexed in PubMed/Medline for its significant contributions to the medical community.

We wanted to acknowledge and provide visibility to Editorial Board members who are deeply involved with the work of *Diseases*, dedicating a Special Issue to their investigations and findings. Therefore, a few months ago, this project spontaneously manifested itself when we discussed the possibility of creating a cutting-edge issue that better represents the values and potentialities of the journal.

This issue is dedicated to this aim, with the awareness that it may represent a useful tool in a plethora of fields in which our Editorial Board members are leading researchers.

There are several growing topics in the current medical and biomedical literature; among them are the potentialities of the application of artificial intelligence [1,2], bioethical concerns regarding its use [3] and regarding palliative care [4], precision medicine [5], new challenges concerning vaccines [6], the use of bioactive compounds as potential adjuvants in therapies [7,8,9,10], gene therapy [11,12,13], etc. Most of these topics have been critically addressed by Dr. Trosko in his enlightening commentary [14], in which he ponders the reliability of interpretations made by artificial intelligence regarding the interaction between our genes and so-called environmental factors, which gives rise to precision therapy for the treatment and prevention of common diseases with a special focus on human carcinogenesis.

Atzori and colleagues [15] focused on another up-to-date concern regarding possible adverse skin reactions to the mRNA SARS-CoV-2 vaccine, reporting a 0.5% prevalence of different mild reactions in a population of Sardinia and characterizing all of them.

A collaborative review between scholars from Pakistan and Italy [16] by De Berardis and colleagues outlined the efficacy of brexanolone, an exogenous analog of allopregnanolone, in the treatment of post-partum depression. Special attention was paid to the cost-related barriers that mainly exist in low-income countries, which mean that it cannot be considered a pharmacological treatment for this growing public health concern that is accessible worldwide.

Finally, a fourth review, which was a systematic review [17], investigated possible risk factors that associate cardiovascular diseases with obstructive sleep apnea, taking into account the fact that hypertension, obesity and diabetes mellitus may largely cause individuals to be at higher risk. Therefore, strategies such as reducing and/or controlling smoking, hypertension, alcoholism, diabetes mellitus, hyperlipidemia and overweight/obesity were suggested.

The research articles collected in this issue dealt with other very interesting topics. For example, the role that lipoprotein (a) level may have in the correct diagnosis of familial hypercholesterolemia is discussed [18]. According to the authors’ findings and their comments, “LDL-C corrected for Lp(a)-cholesterol should be considered in all FH patients with Lp(a) level above 40 mg/dL for recalculating points in accordance with DLCN criteria”.

Hospitalizations represent one of the higher social costs for our national health systems; therefore, controlling costs is an ever-increasing challenge that should be addressed with better diagnoses and better outcomes. In this sense, Gangu et al. [19] addressed evidence concerning the worst outcomes and increased related costs when biliary tract diseases are not adequately treated.

As previously discussed at the beginning of this Editorial, artificial intelligence is one of the most cutting-edge topics in medicine, and machine learning may emerge as one of the most promising fields of application in this discipline. For example, patients with hyponatremia may be classified in different risk clusters by means of a machine learning approach which does not require human supervision [20]. Authors were able to identify three clinically distinct phenotypes with differing mortality risks in a heterogeneous cohort of hospitalized hyponatremic patients, which contributed greatly to the awareness that this kind of approach may be extremely useful in daily hospital activities.

Finally, an interesting approach was used by Saadeddine and colleagues [21] to set up protocols and tests for the evaluation of strength and performance, in a screening mode, of skeletal muscle mass reduction, which is a quite common event in older adults during a plethora of diseases and is associated with longer periods of hospitalizations which, in turn, cause increased costs, and of course, increased mortality rates.

Overall, the studies outlined above are the first, very useful contributions of our Editorial Board members which represent the pillars for a new Special Issue collection to be executed by other colleagues in the forthcoming years.
